# The Swedish National Formulary for Children ePed. Comment on Shaniv et al. A Callout for International Collaboration. Reply to Giger, E.V.; Tilen, R. Comment on “Shaniv et al. Neonatal Drug Formularies—A Global Scope. *Children* 2023, *10*, 848”. *Children* 2023, *10*, 1803

**DOI:** 10.3390/children11111339

**Published:** 2024-10-31

**Authors:** Christiane A. Garnemark, Per Nydert, Synnöve Lindemalm

**Affiliations:** The Swedish National Formulary for Children ePed, Astrid Lindgren Children’s Hospital, Division of Paediatrics, Karolinska University Hospital, 171 76 Stockholm, Sweden; per.nydert@regionstockholm.se (P.N.); synnove.lindemalm@regionstockholm.se (S.L.)

We have read with interest both the article by Shaniv D. et al. entitled “Neonatal Drug Formularies-A Global Scope” [1] and the following response to Giger, E.V.; Tilen, R. “A Callout for International Collaboration” [2] and want to respond to the callout for international collaboration and add information about the Swedish National Formulary for Children.

Paediatric and especially neonatal pharmacotherapy often requires the use of unlicensed and off-label medicines [3,4]. The off-label concept is closely connected to the pharmaceutical industries perspective. ePed starts from the user’s perspective and tries to fill the users’ gaps towards off-label use. In Sweden, there is an awareness of off-label use in paediatric medicine and the regulatory authorities work together with ePed for a safer use of medications in children. The primary aim of the Swedish National Formulary for Children ePed is to provide practical drug information for healthcare professionals to safely order, prepare and administer these medicines [5]. The approach is unique in that the database provides information on drug doses as well as information on preparation and administration. Based on clinical need, evidence and experience-based information are compiled from a variety of sources. The information is updated and reviewed weekly through discussion with numerous experts within Swedish paediatric healthcare and in close cooperation with all children’s hospitals as well as the Swedish Paediatric Society.

The Swedish National Formulary for Children ePed started in 2005 as a database for medicines in the neonatal units in Stockholm, where the Sachsska and Astrid Lindgren Children’s Hospitals wanted to collaborate. The work was soon extended to all children aged 0–18 years, and thus to all paediatric healthcare in Stockholm. Since 2014, ePed has become a national decision support tool that is funded nationally, on a joint basis by all Swedish regions. As a national decision support tool, ePed has been integrated into the various electronic health record (HER) systems used in Sweden, making the information easily accessible. The central editorial office at Astrid Lindgren Children’s Hospital at Karolinska University Hospital (ALB) manages and develops ePed on an ongoing basis. The widespread use of ePed has created a collaborative system that shares information about medication treatment in a unique network between children’s hospitals in Sweden. The willingness to collaborate and the understanding of the benefits of co-creating standardisation is an additional benefit. At the same time, reaching a consensus is sometimes a challenge that requires enormous effort. If we can not reach a consensus, we present the different practises or dosages in the ePed instruction. In this way, we can show the lack of a consensus, and the information can be used for an ongoing discussion to reach a national consensus.

With the growing international interest in ePed, we are faced with the challenge of translating the information into English. The ePed database is developed for Swedish use and adapted to Swedish healthcare traditions. We are aware that there are differences between countries based on legislation, digital infrastructure, treatment traditions and different responsibilities between the professions involved in the pharmaceutical process. Simply translating the information in ePed may lead to incorrect results. Adapting the ePed database for international use is a major challenge for the future.

Today, the ePed database contains paediatric medication information through more than 1200 ePed instructions including 430 substances according to the Anatomical Therapeutic Chemical (ATC) classification system. For more than 230 substances, there is a dose range check with warning for an under/overdose for individual and total daily doses available. In 2023, 61 new ePed instructions were published, and 639 existing ePed instructions were updated (with 254 major and 379 minor updates) [5].

The initiative to update an ePed instruction is taken when there is a need for change, e.g., due to comments from clinical users or a need to update the information due to a newly registered or temporarily unavailable medication. Information on medication errors from all paediatric hospitals in Sweden also leads to changes in ePed instructions where information is clarified or a dose range check is added to prevent future errors. Every week, an inter-professional group consisting of a paediatrician, a paediatric nurse and a pharmacist review all medication errors at Astrid Lindgren’s Children’s Hospital. This work is influencing the work with ePed instructions and contributes to understanding the challenges of paediatric medicines and improving patient safety.

We appreciate the initiative of the authors to collect information on neonatal drug formularies, as premature infants are a patient group where information on drug doses is scarce and where collaboration is particularly needed to provide the safest and best possible care for this vulnerable group of patients. However, we also recognise the need for reliable drug information for other paediatric patients. Below, we would like to provide you with information on the Swedish paediatric formulary ePed according to the questionnaire in the Supplementary Material of the original paper [1].

## 1. Questionnaire for the Formularies, Version 3.2, 19 October 2022

### 1.1. When Was the Formulary First Published?

ePed started in 2005 as a database for medicines in the neonatal units in Stockholm, where the Sachsska and Astrid Lindgren Children’s Hospitals wanted to collaborate. A database was created to generate lists of medicines and dilution schemes. The work was soon extended to all children from 0 to 18 years, and thus to all paediatric care.

The information can be found on www.eped.se, accessed on 28 May 2024 [5], but the information is also integrated to existing electronic health record systems through the Swedish information services for drugs (SIL) [6], where it is directly connected to medication orders and can be easily accessed by doctors, registered nurses and pharmacists caring for children in Sweden. Each Swedish region has its own regional ePed committee, which reviews and then selects which ePed instructions are accepted locally.

### 1.2. What Format(s) Is It Published In?

- Web-based: information can be found on www.eped.se [5] but the information is also integrated through the Swedish information services for drugs (SIL) [6] to medication orders within the different electronic health record systems used in Sweden.
- Mobile application: you can access the information through www.eped.se [5] on your mobile phone.
- Print: there is no printed version available since there are weekly updates.

### 1.3. Who Is the Target Audience?

All doctors, registered nurses and pharmacists who care for children in hospitals and primary care in Sweden. Each Swedish region has its own regional ePed committee, which reviews and comments on the ePed instructions within a collaborative software, Centeped (version 2.5), provided through the Swedish information services for drugs (Sil) [6]. This results in a national consensus process of best practises for safer use of paediatric medications.

### 1.4. What Language(s) Is It Published In?

Swedish; there are ongoing discussions about translation into other languages, e.g., English.

### 1.5. Is It Linked to a Broader Formulary (Adults, Children) or Is It a Standalone Formulary?

The formulary is currently available for neonatal and paediatric patients up to 18 years of age. There is an ongoing development of the formulary, primarily for adults in intensive care.

The ePed formulary includes the following:

- Indication and dose information.
- Information on how to reconstitute and administer the medicine, as well as important additional information on storage and shelf life.
- A dose range check with over/underdose warnings for single and daily doses.
- Links to references used in the ePed instruction, including links to national or international treatment recommendations and relevant published studies.
- Information on best practice in the preparation of medicines. Based on Council of Europe Resolution CM/Res (2016)2 [7].

### 1.6. What Is the Business Model?

Since 2014, ePed has been a national decision support tool financed by national funds on a joint basis by all Swedish regions. The information is distributed via the Swedish information services for drugs (SIL) [6] to electronic health record systems and is supported by an editorial office consisting of two paediatricians (whom have additional subspecialties in clinical pharmacology and neonatology), one paediatric nurse and nine pharmacists specialising in paediatrics.

### 1.7. What Is the Frequency of Updates?

The information is published weekly and managed electronically by a central procedure through the collaborative software Centeped, which allows the generated drug information to be published with version control in a national electronic library with metadata. Centeped (ePed database) also acts as a gatekeeper, where the published information is implemented into electronic health record (HER) systems by different Swedish healthcare regions, only after regional acceptance. Each central major version update must be re-accepted before it is implemented regionally.

- Are there “major” updates and “minor” updates?
  There are both minor and major updates. Major updates must be approved locally by the regional ePed committee. Minor updates need no new approval from the regional ePed committee to be available in their electronic health record system.
- Are updates always scheduled, or does new evidence lead to an immediate update?
  An ePed instruction is updated when there is a need to do so. This may be due to a desire for a new indication, additional information, or the need to update information that needs to be made available to users, e.g., new information on preparation or administration, new available drugs or changes due to drug shortage.
  Updates are usually made on Thursdays but can be made immediately at any time, if necessary, directly.

### 1.8. How Are Updates Communicated to Users?

All updates are published in an open format on www.eped.se [5]. Each regional ePed committee has access to Centeped (the ePed database) and decides which ePed instructions are to be used locally. If there has been a major update to a regionally selected ePed instruction, it must be approved locally. There is an opportunity to comment to the national ePed committee if there are local differences to the information provided in the ePed instruction. This opportunity for comment and discussion has helped to achieve national consensus for many drugs.

### 1.9. How Are Drugs Selected for Inclusion?

- How are drugs first identified for possible inclusion?
  The drugs in the database are identified by their clinical use and the need for clinically relevant information. The national ePed committee receives “requests” for a new ePed instruction from the regional ePed committees. Known medication errors also lead to new or revised ePed instructions.
- How is it determined when there is enough need and/or evidence for inclusion?
  If there is a clinical need for an ePed instruction, the national ePed committee will determine if reliable information is available and decide whether or not to produce an ePed instruction. Even for medications that are rarely used, there may be a need for an ePed instruction. Especially when information is difficult for the individual doctor or nurse to find, it is a patient safety issue to have reliable information collected and presented for users. If we have a dose recommendation and the evidence for that dose is low, we mark the indication as ‘low evidence’. This tells the prescribing doctor that there are few trials for this indication or group of patients and that the patient should be monitored closely for the risk of side effects.

### 1.10. Who Is Involved in the Development of a Monograph?

- Expertise
  The national ePed committee consists of two paediatricians (with sub-specialisation in clinical pharmacology and neonatology), one paediatric nurse and nine pharmacists specialising in paediatrics.
- Hierarchy of decision making
  The information gathered through the dose research process is discussed within the members of the national ePed committee together with doctors (mainly paediatricians and subspecialists) from all over Sweden.
  Each region reviews the ePed instruction prior to approval. Comments from the regional ePed committees are received and new dialogue and discussion may be necessary until a national consensus is reached. There are always ongoing discussions about doses and treatment recommendations. This is a work in progress that is carried out together with the Swedish Paediatric Society, its subspecialty groups and other experts with medical specialties such as anaesthesia, infectious diseases, etc. Final approval is given by the national ePed committee before the ePed instruction is published.
  The national ePed committee has regular contact with several Swedish authorities, e.g., the Swedish National Board of Health and the Swedish Medical Products Agency, and is a valued knowledge resource for paediatric pharmacological issues at a national level.

### 1.11. What Is the Search Process for Literature Support?

Dose research is conducted to gather information provided in local instructions and known formularies, e.g., BNF-C, NeoFax/Micromedex, Neonatal Formulary and UpToDate. In addition, PubMed and Cochrane are searched for published articles and reviews on the substance in neonatal and paediatric patients. The information gathered is discussed in an interdisciplinary manner with physicians specialising in paediatrics, neonatology and paediatric pharmacology; paediatric and neonatal nurses; and pharmacists.

- Are specific PubMed terms used on all drugs?
  The PubMed search is performed according to the need for information, e.g., a specific diagnosis or time for drug administration.
- Is there a “deeper dive” into references listed in the initial articles retrieved?
  We show the main references in the ePed instruction if they are important for specific information in the text. There is a background document with more complete information and references that is not published.
- What is considered “best evidence”?
  We always discuss treatment suggestions with specialists in the specific field as well as the Swedish Medical Products Agency. We also publish information with low evidence if this is necessary for clinical use, but the information is marked as “low evidence”.
- How are expert panel recommendations utilised?
  If there is a need for an expert panel, we recruit stakeholders from the area of expertise within the Swedish Paediatric society or its subspeciality groups.

### 1.12. Is the Dosing Information Evaluated Differently than Other Sections of the Monograph?

- If there is not a single standard dose, are multiple doses mentioned versus a range?
  Yes, we present doses according to indications and if needed a range is presented.
  Sometimes, the more “unusual” doses are presented in parentheses.

### 1.13. Are Extrapolations Carried Out from PK/PD Data in Children?

This is only conducted in very rare cases when other data are missing.

### 1.14. How Is Product Labelling Used in Constructing the Drug Monograph?

- What if the labelling does not mention neonates?
  If we find information in other sources about doses for neonates, we will present it, even if it is off label.
- What if the labelling has a vague warning about use under a certain age?
  If the warning is relevant, we include it in the ePed instruction. Age-specific explanations are used; for example we inform on the reason for the drug having a certain age limit and how to handle the risk, e.g., drugs containing benzyl alcohol.
- How are black box warnings included in the monograph?
  We do not use the full US black box warnings but we have a warning in the ePed instruction for high-risk situations which can include warnings for all types of risks. In addition, we have a risk assessment tool in “best practice” with a four-step warning sign allowing ePed to highlight situations related to work environment (e.g., antibiotics), reconstitution risks (e.g., two-step dilutions), narrow therapeutic windows (e.g., digoxin) and microbiological risks (e.g., intravenous fat emulsions).
